# Bio-Engineered Nisin with Increased Anti-*Staphylococcus* and Selectively Reduced Anti-*Lactococcus* Activity for Treatment of Bovine Mastitis

**DOI:** 10.3390/ijms22073480

**Published:** 2021-03-27

**Authors:** Des Field, Kiera Considine, Paula M. O’Connor, R. Paul Ross, Colin Hill, Paul D. Cotter

**Affiliations:** 1 School of Microbiology, University College Cork, T12 K8AF Cork, Ireland; p.ross@ucc.ie; 2 APC Microbiome Ireland, T12 YT20 Cork, Ireland; p.cotter@teagasc.ie; 3 Teagasc Food Research Centre, Moorepark, Fermoy, Co., P61 C996 Cork, Ireland; kiera.m.considine@gmail.com (K.C.); paula.oconnor@teagasc.ie (P.M.O.)

**Keywords:** antimicrobial, lantibiotic, bacteriocin, peptide engineering, nisin, bovine mastitis, staphylococci, *S. aureus*

## Abstract

Bovine mastitis is a significant economic burden for dairy enterprises, responsible for premature culling, prophylactic and therapeutic antibiotic use, reduced milk production and the withholding (and thus wastage) of milk. There is a desire to identify novel antimicrobials that are expressly directed to veterinary applications, do not require a lengthy milk withholding period and that will not have a negative impact on the growth of lactic acid bacteria involved in downstream dairy fermentations. Nisin is the prototypical lantibiotic, a family of highly modified antimicrobial peptides that exhibit potent antimicrobial activity against many Gram-positive microbes, including human and animal pathogens including species of *Staphylococcus* and *Streptococcus*. Although not yet utilized in the area of human medicine, nisin is currently applied as the active agent in products designed to prevent bovine mastitis. Over the last decade, we have harnessed bioengineering strategies to boost the specific activity and target spectrum of nisin against several problematic microorganisms. Here, we screen a large bank of engineered nisin derivatives to identify novel derivatives that exhibit improved specific activity against a selection of staphylococci, including mastitis-associated strains, but have unchanged or reduced activity against dairy lactococci. Three such peptides were identified; nisin A M17Q, nisin A T2L and nisin A HTK.

## 1. Introduction

Bovine mastitis is a serious disease involving inflammation of the mammary gland that affects dairy cattle worldwide. It is the most frequent infectious disease among dairy cattle, costing the US dairy industry an estimated USD 2 billion annually [1]. Microorganisms are the causative agents for the inflammatory reaction, and *Staphylococcus aureus* is thought to be accountable for 15–30% of the infections [2,3]. The treatment of mastitis with antibiotics during lactation is customary, given that between 2% and 55% of cows are mastitic during this period [4]. Notably, *S. aureus*-associated infection is categorized by significantly lower cure rates compared to mastitis caused by other bacteria, mainly due to the regular acquisition of antibiotic resistance determinants or resistance-facilitating mutations by the pathogen [5,6]. Indeed, chronic *S. aureus* intramammary infections are one of the most common reasons for premature culling in dairy herds [7]. The issue is further compounded through antibiotic use to treat animals destined for human consumption by selecting for antibiotic resistance development among food microorganisms and by the potential exposure of consumers to antibiotic residues in milk and dairy products [8]. In addition, trace levels of antibiotics in milk destined for fermented food manufacture may inhibit the growth of and fermentation by bacterial starter cultures and result in products of insufficient quality [9]. Similarly, such antibiotic residues can negatively impact the other diverse range of microbes present in unpasteurised milk and countless dairy products that play important roles in fermented food production by contributing to flavour and aroma development, food safety, or providing several potential health or nutritional benefits to the consumer [10].

While the complete elimination of antibiotic therapy for mastitis is unlikely given modern intensive farming practices, a reduction in antibiotic use is highly desirable. One group of compounds under consideration as a potential alternative are the lantibiotics. Lantibiotics are a class of bacteriocins (bacterially derived ribosomally synthesized antimicrobial peptides [11,12,13]) that are characterised by the presence of posttranslationally modified amino acids including lanthionine and/or methyllanthionine [14,15]. The best known lantibiotic is nisin A. Produced by some strains of *Lactococcus lactis,* nisin is active against a wide range of Gram-positive bacteria, including foodborne pathogens from the genera *Staphylococcus*, *Bacillus* and *Clostridium*. Nisin is generally regarded as safe and has both European and US Food and Drug Administration (FDA) approval for use as a food preservative [16]. Moreover, the original nisin A, and its natural derivative nisin Z, demonstrate efficacy against the Gram-positive aetiological agents responsible for bovine mastitis and numerous studies have explored the use of nisin-based formulations to control or treat such infections [17,18,19]. In particular, in a field trial consisting of 139 cows with subclinical mastitis, Mast Out, a nisin-based treatment was effective in curing mastitis caused by several different pathogens [19].

Over the last decade or so, the application of bioengineering approaches has established that nisin functionality can be improved through as little as a single residue change [20,21,22,23]. In addition to being fundamentally interesting, these derivatives also have the potential to be applied in a variety of ways as a consequence of superior activity against pathogenic bacteria including mastitis-associated *S. aureus* and *S. agalactiae* [24,25], as well as many drug-resistant microbes including methicillin-resistant *S. aureus* (MRSA), vancomycin-intermediate *S. aureus* (VISA), coagulase-negative staphylococci and *Staphylococcus pseudintermedius* [26,27]. Notably, nisin can also be engineered to provide protection against the nisin resistance protein [28] that acts through the enzymatic cleavage of nisin [29]. Importantly, it was also apparent from these studies that several bioengineered nisin derivatives exhibit strain- or species-specific enhanced potency. In view of the high therapeutic potential of nisin as an anti-mastitis agent, coupled with the aforementioned capacity to be bioengineered, the aim of this study was to implement a screening strategy utilising both mastitis-associated organisms and representative ‘beneficial’ milk-associated lactic acid bacteria (LAB) with the ultimate aim of isolating derivatives of nisin with activity that was enhanced against the pathogenic microbes but not against LAB. The strategy proved successful in that three previously uncharacterized nisin variants were identified that exhibit improved specific activity against a selection of mastitis-associated staphylococci but with equal, or in some cases, reduced activity against several LAB indicators when compared to the wild-type nisin A control. 

## 2. Results

### 2.1. Screening of nisin Derivatives for Enhanced Antimicrobial Activity Against S. aureus Strains Associated with Bovine Mastitis

Previously, site-saturation mutagenesis strategies were used to generate bioengineered nisin derivatives in which all residues not involved in ring formation (i.e., all residues other than serines, threonines and cysteines involved in the lanthionine ring A and methyllanthionine rings B, C, D and E) were randomised to potentially all other natural amino acids, or in which all three residues of the hinge-region (NMK) were simultaneously randomised [20,24,30]. Here, these combined banks of almost 30,000 independent producers were screened using deferred growth inhibition assays to identify those which displayed greater bioactivity (as assessed by zones of clearing) than nisin A against mastitis-associated staphylococci (*S. aureus* RF122, *S. aureus* NCDO1499) and streptococci (*S. agalactiae* ATCC13813), but were not enhanced against the LAB strains *L. lactis* HP and *L. lactis* MG1363 (data not shown). From this screen, 12 variants of interest from the first round of screening were incorporated into a “mini-bank” for subsequent further analysis. The number of indicator strains was increased to include bovine mastitis-associated *S. aureus* DPC 5243, *S. dysgalactiae* ATCC43078, *S. uberis* DPC5344, as well as other LAB organisms such as *L. lactis* IP5, *L. lactis* sp *cremoris* KH, and *Lb. acidophilus* ATCC4356 (Table 1).

**Figure 1:**
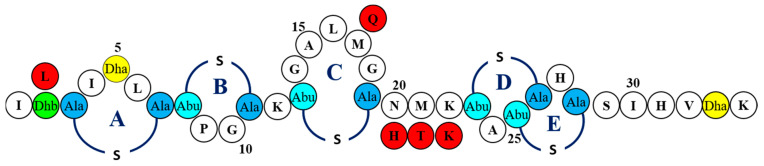
Primary structure of nisin A. Residues are represented in the single letter code. Post translational modifications are indicated as follows, Dha: dehydroalanine, Dhb: dehydrobutyrine, Abu: 2-aminobutyric acid, Ala-S-Ala: lanthionine, Abu-S-Ala: 3-methyllanthionine. Amino acid substitutions nisin A T2L, nisin A M17Q and nisin A HTK are highlighted in red.

Following this second round of screening, three variants of interest were selected for closer inspection based on their contrasting zones of inhibition against the pathogenic and LAB targets. DNA sequencing of the mutated nisin gene as well as mass spectrometric evaluation of the producing strain established that these corresponded to nisin A M17Q (methionine at position 17 altered to glutamine), nisin A T2L (threonine 2 leucine) and nisin A HTK (where the hinge residues Asn-Met-His have been altered to His-Thr-Lys) (Figure 1). The nisin A M17Q producing strain was notable by virtue of the fact that it displayed the greatest bioactivity towards the majority of the pathogenic indicators compared to the other nisin variants of this study, although this enhanced activity was also apparent against three of the LAB strains (Table 1).

In the case of the nisin A HTK producing strain, the bioactivity appeared enhanced against the panel of staphylococci (*S. aureus* RF122, *S. aureus* NCDO1499 and *S. aureus* DPC 5243) but was distinguished by zones of inhibition that were similar to the wild type nisin A against the LAB targets (Table 1). In contrast to the nisin A M17Q and nisin A HTK producing strains, the nisin A T2L producer did not exhibit visibly enhanced activity against any of the pathogenic bacteria utilised in this screen, but was noteworthy in that the bioactivity appeared reduced against the panel of lactococci and lactobacillus strains (Table 1). Given the distinctive bioactivity profiles of the nisin variants and the aim of the study to identify enhanced anti-*S. aureus* derivatives, we examined several other strains, including two methicillin resistant *S. aureus* (MRSA), ST528 and ST534, as well as *S. aureus* SA113 and the mutants *S. aureus* SA113Δ*mpr*F and *S. aureus* SA113Δ*dlt*A, using deferred antagonism assays. The results were consistent with the previous observations, whereby the strain producing nisin A M17Q again displayed the greatest zones of inhibition, followed by nisin A HTK and nisin A T2L, with nisin A T2L exhibiting equal activity to the wild type nisin A producing control. On this basis, the derivatives nisin A M17Q, nisin A T2L and nisin A HTK were purified by HPLC (Figure S1) for specific activity determination. Purified peptides were subjected to MALDI TOF mass spectrometry to confirm the correct mass in each case (Figure S1). In the case of the nisin A HTK derivative, the threonine remained in the unmodified form, i.e., in a manner consistent with previous observations [24] relating to the derivative nisin A M21T.

### 2.2. MIC-Based Investigations Demonstrate Enhanced Specific Activity of nisin Derivatives against Bovine Mastitis-Associated S. aureus

MIC assays were carried out using equimolar concentrations of wild type nisin A, nisin A M17Q, nisin A T2L, and the hinge variant nisin A HTK against a range of targets together with 12 *S. aureus* isolates including bovine-mastitis associated strains RF122, NCDO1499, DPC 5243, DPC 5245, DPC 5247, as well as *S. aureus* isolates 513, 272, SA113, SA113Δ*mpr*F, *S. aureus* SA113Δ*dlt*A and two isolates of methicillin-resistant *S. aureus* (MRSA) ST 528 and ST 534. Lactic acid bacteria targets *L. lactis* MG1363, *L. lactis* HP, *L. lactis* KH and *Lb acidophilus* ATCC 4356 as well as the *Bifidobacterium* strain *B. longum* UCC44b were also included. The MIC was determined to be the lowest concentration of peptide that resulted in the absence of visible growth of the target strain after 16 h at 37 °C. All three nisin derivatives displayed different levels of activities against the panel of *S. aureus* strains tested. Nisin A M17Q displayed enhanced specific activity against four of the *S. aureus* strains utilised in the study. The mean MIC value for nisin A against the mastitis-associated *S. aureus* NCDO1499 and *S. aureus* RF122 strains was 2 µg/mL (625 nM) and 1 µg/mL (312 nM), respectively (Table 2). In contrast, the corresponding nisin A M17Q MIC values were 1 µg/mL (312 nM) and 0.25 µg/mL (78 nM), respectively, representing a 2 and 4-fold increase in potency, respectively. Nisin A M17Q also displayed 2-fold activity against *S. aureus* 272 and MRSA ST528 (Table 2). However, the specific activity of nisin A M17Q remained equal to that of wild type nisin A against the remaining 7 *S. aureus* targets despite the observed larger zones of inhibition generated by the derivative producer against these strains (Table 1). In the case of the streptococcal target *S. dysgalactiae* ATCC43078, nisin A M17Q proved to be 2-fold less active than the wild type (8 µg/mL and 4 µg/mL, respectively).

The nisin A HTK derivative also displayed superior potency compared to nisin A against *S. aureus* NCDO1499 (1 µg/mL) and *S. aureus* RF122 (0.12 µg/mL), representing a 2 and 8-fold increase in potency, respectively (Table 2). While the derivative also proved 2-fold more effective against ST528 (MRSA), it was not more effective against the staphylococcal targets DPC 5243, DPC 5245, DPC 5247, as well as *S. aureus* isolates 513, 272, SA113, SA113Δ*mpr*F, *S. aureus* SA113Δ*dlt*A and *S. aureus* ST 534 (MRSA).

Lastly, although nisin A T2L exhibited comparable bioactivity as the parental nisin A against the panel of pathogenic organisms in deferred antagonism assays, the activity of the purified peptide was particularly notable as it displayed improved activity against all of the *S. aureus* strains utilised. Notably, T2L exhibited 2-fold activity against *S. aureus* DPC5243, 5245, 5247 and 513, 4-fold enhanced activity against *S. aureus* RF122, 272, ST528 and 534 (MRSA) and 8-fold activity against SA113. When the SA113 mutant strains were examined, nisin A T2L displayed 16-fold improved specific activity against SA113Δ*mpr*F (Table 2) compared to nisin A (4 and 0.25 µg/mL, respectively) and was also 16-fold more efficacious against *S. aureus* SA113Δ*dlt*A than wild type (2 and 0.06 µg/mL, respectively) (Table 2).

When the lactococcal targets were assessed, the nisin variants again exhibited differing specific activities depending on the target strain employed. Against *L. lactis* MG1363, both nisin A HTK and nisin A M17Q proved to be 2-fold more potent than nisin A (0.1, 0.1 and 0.2 µg/mL, respectively), while the MIC of nisin A T2L remained equivalent to the wild-type peptide (Table 2). In contrast, nisin A HTK and nisin A T2L displayed a two-fold reduction in potency (0.4 µg/mL) against *L. lactis* HP (Table 2) whilst nisin A M17Q was 2-fold more potent (0.1 µg/mL) compared to wild type nisin A (0.2 µg/mL). When *L. lactis* KH was assessed, both nisin A HTK and nisin A M17Q displayed MIC values equivalent to the wild type nisin A peptide (0.05, 0.05 and 0.05 µg/mL, respectively) but nisin A T2L exhibited 2-fold less potency. Against the target strain *B. longum* UCC 44b, both nisin A HTK and nisin A T2L displayed four-fold (0.24 µg/mL) and two-fold (0.12 µg/mL) decreased activity, respectively, compared to wild type peptide (0.06 µg/mL, while nisin A M17Q displayed equal activity to nisin A. Finally, when *Lb. acidophilus* ATCC 4356 was employed as the target, nisin A HTK and nisin A T2L exhibited a four-fold reduction (0.12 µg/mL) in specific activity compared to wild type nisin A (0.03 µg/mL) and, in this case, nisin A M17Q was two-fold less active (0.06 µg/mL).

These results highlight the disparate sensitivity of different species and strains within the same species to antimicrobial peptides such as nisin, and the requirement to utilize a broad range of test organisms in order to achieve the greatest insight into its antimicrobial potential such that a peptide of interest is not overlooked.

### 2.3. Growth Curve Based Assessment

To provide more insight into the inhibitory effects of nisin A HTK, nisin A M17Q and nisin A T2L, the peptides were further investigated by means of growth curve experiments. For growth curves, we included the bovine pathogen *S. aureus* RF122 and a strain of *S. aureus* (SA113) often utilised as an archetypal staphylococcal organism in model virulence studies [31]. The lactic acid bacteria were represented by two lactococcal strains, *L. lactis* subsp *cremoris* KH and *L. lactis* subsp *cremoris* IP5. 

**Figure 2:**
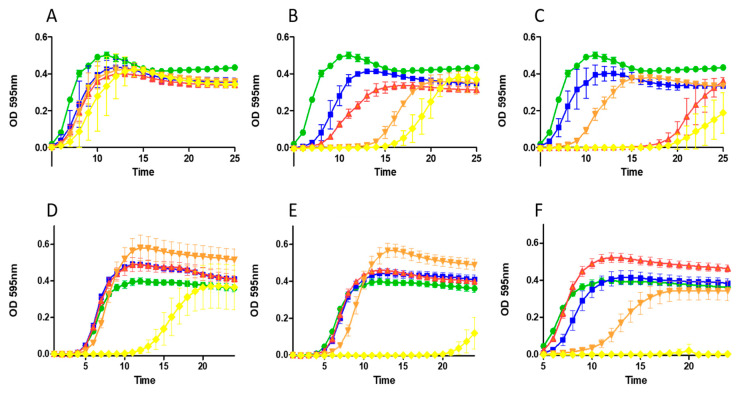
Impact of increasing concentrations of nisin A and nisin derivatives on growth of staphylococci. Effect of nisin A (blue square) and nisin variants nisin A M17Q (orange triangle), nisin A HTK (red triangle) and nisin A T2L (yellow diamond) and untreated control (green circle) on *S. aureus* RF122 in 0.32 µg mL^−1^ (**A**), 0.48 µg mL^−1^ (**B**) and 0.64 µg mL^−1^ (**C**) and *S. aureus* SA113 in 3.2 µg mL^−1^ (**D**), 4.8 µg mL^−1^ (**E**) and 6.4 µg mL^−1^ (**F**), The means and standard deviations of three independent determinations are presented.

*S. aureus* RF122 was treated with a range of peptide concentrations (Figure 2) and, in each case, the results were consistent with the increased potency of nisin A HTK, nisin A M17Q and nisin A T2L compared to the parental nisin A and in agreement with MIC data. 

At 150 nM (0.48 µg/mL) (Figure 2B) and 200 nM (0.64 µg/mL) (Figure 2C), all three derivatives brought about a protracted lag phase of growth with nisin A T2L exhibiting the greatest potency. Similarly, when *S. aureus* SA113 (Figure 2) was assessed, the most potent peptide observed was nisin A T2L, though nisin A M17Q appeared to show evidence of heightened inhibitory effects compared to either nisin A HTK or nisin A at 1.5 µM (4.8 µg/mL) (Figure 2E) and 2.0 µM (6.4 µg/mL) (Figure 2F) concentrations highlighting the more subtle effects on growth inhibition which are often missed in end point assays such as MIC experiments.

**Figure 3:**
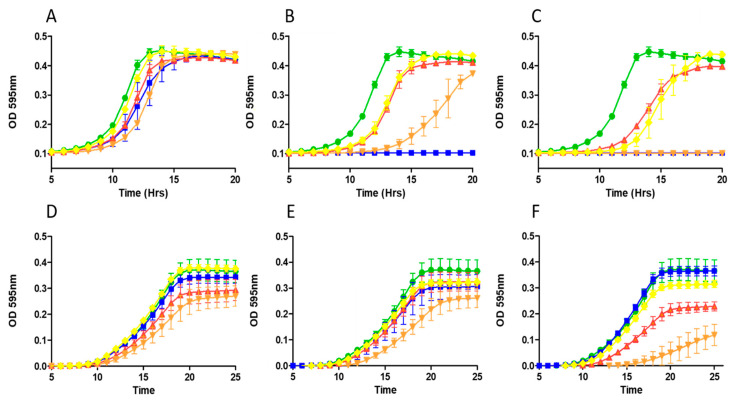
Impact of increasing concentrations of nisin A and nisin derivatives on growth of lactococci. Effect of nisin A (blue square) and nisin variants nisin A M17Q (orange triangle), nisin A HTK (red triangle) and nisin A T2L (yellow diamond) and untreated control (green circle) on *L. lactis* KH in 0.14 µg/mL (**A**), 0.32 µg/mL (**B**) and 0.64 µg/mL (**C**) and *L. lactis* IP5 in 0.16 µg/mL (**D**), 0.32 µg/mL (**E**) and 0.48 µg/mL (**F**). The means and standard deviations of three independent determinations are presented.

When *L. lactis* subsp *cremoris* KH was challenged with increasing levels of nisin A and nisin derivatives, all three variants proved to be less active compared to nisin A, again highlighting the distinct difference relative to activity against staphylococcal strains. While no significant difference was observed in the growth of *L. lactis* KH at the lowest test concentration (45 nM) (Figure 3A), nisin A appeared to completely inhibit growth at 100 nM (0.32 µg/mL) (Figure 3B).

In contrast, both nisin A HTK and nisin A T2L proved to be less active as observed by a shorter lag time, though nisin A M17Q did produce a more pronounced inhibitory effect (Figure 3B). Indeed, a further increase in peptide concentration (200 nM; 0.64 µg/mL) brought about complete inhibition of growth by nisin A M17Q and nisin A but not by nisin A HTK or nisin A T2L (Figure 3C). When *L. lactis* subsp *cremoris* IP5 was challenged, nisin A HTK and nisin A M17Q proved more potent with increasing doses of peptide than either nisin A or nisin A T2L, which exhibited similar growth profiles (Figure 3D–F).

### 2.4. Impact of Derivatives on Nisin Induction and Production

**Figure 4:**
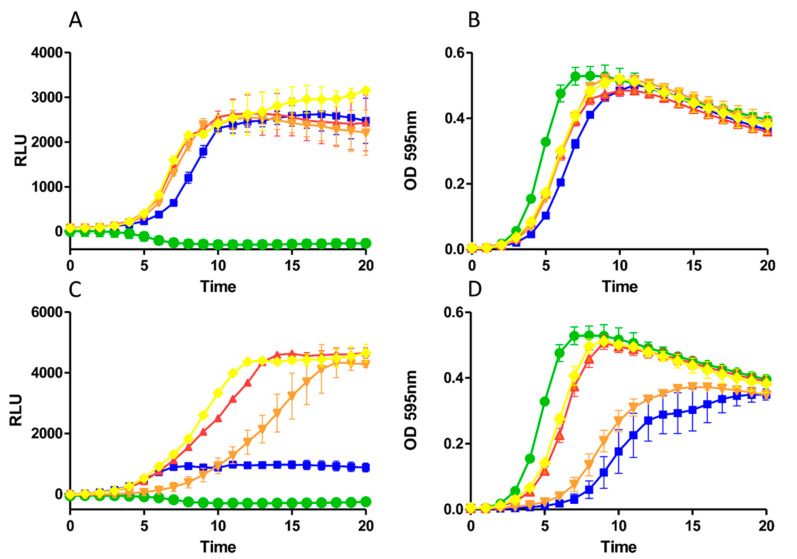
Comparison of induction capacities of nisin A (blue square), nisin A M17Q (orange triangle), nisin A HTK (red triangle), nisin T2L (yellow diamond) and untreated control (green circle) as determined by expression of GFP under the control of the P*nisA* promoter in *L. lactis* NZ9000 pNZ8150*gfp+* induced at final concentrations of (**A**) 10 ng/mL and (**C**) 30 ng/mL and the effects on growth of *L. lactis* NZ9000 pNZ815*0gfp+* induced at concentrations of (**B**) 10 ng/mL and (**D**) 30 ng/mL as determined by absorbance at OD_595nm._

The contrasting bioactivity of nisin A T2L as observed in deferred antagonism assays compared to MIC and growth curve assays against the range of staphylococci indicators tested was unanticipated. Accordingly, we sought to investigate if peptide production or induction capacity was negatively impacted as a result of the newly introduced residues in nisin A HTK, nisin A M17Q and nisin A T2L. To that end, the induction capacities of the variant peptides were determined using a GFP reporter strain [28,32] at 10 ng/mL, which reflects the commonly used concentration for nisin induction, but also at 30 ng/mL which has proved to be inhibitory to the host strain *L. lactis* NZ9000 [32]. Additionally, growth of the strains was monitored in parallel and recorded as absorbance readings (OD_595_). No significant variation in fluorescence (as measured in relative light units [RLU]) was observed for any of the derivatives when compared to wild type and induced at 10 ng/mL (Figure 4A).

Indeed, the rate of expression, and thus induction, appeared identical for all peptides tested with a maximum intensity achieved after 10 h. Similarly, little impact on growth of the strains at this peptide concentration was observed when compared with the uninduced control (Figure 4B) and was in agreement with previous findings [32]. However, when the higher concentration of 30 ng/mL was applied, a significant impact on the rate and level of GFP expression was detected for nisin A (Figure 4C). In contrast, the variant peptides induced higher intensities of fluorescence than at 10 ng/mL, though the rate was more elongated and the maximum intensity achieved was at 12, 14 and 18 h for nisin A HTK, nisin A T2L and nisin A M17Q, respectively. When the growth profiles of the strains were examined, nisin A proved inhibitory to growth as evidenced by a lengthy lag phase (Figure 4D). The M17Q derivative also elicited a delay in bacterial growth, which coincided with delayed expression of GFP. In contrast, it was clearly evident that both nisin A HTK and nisin A T2L had only a minimal impact on the growth and consequently GFP expression of the reporter strain, further supporting previous MIC and growth curve outcomes with the other lactococcal strains tested.

## 3. Discussion

The commercial significance of mastitis as a persistent disease in dairy farming coupled with the emergence of antibiotic resistance in pathogenic bacteria has spurred exploration into the most advantageous treatment strategies for current antimicrobial compounds as well as a search for new alternatives to conventional therapy. Due to its distinctive mode of action, broad range of antimicrobial activity and its gene-encoded nature, nisin A is an obvious choice for genetic manipulation in a bid to enhance its functionality. Indeed, several recent bioengineering studies involving nisin and other lantibiotic peptides, including mutacin, mersacidin, lichenicidin, and nukacin ISK-1, have been successful in that regard as a consequence of the creation and screening of substantial banks of engineered peptides [24,33,34,35]. In this study, we undertook the largest screen of such peptides to date involving approximately 30,000 nisin derivatives against several representative mastitis-associated pathogenic targets including staphylococci and streptococci. We also set ourselves the more difficult task of uncovering novel derivatives with two distinct properties, namely to demonstrate enhanced activity towards pathogenic targets and show reduced activity towards many of the commensal organisms naturally present in milk such as lactococci and lactobacilli. In this regard we were successful in that three new nisin variants, nisin A M17Q, nisin A T2L and nisin A HTK, exhibited these characteristics, albeit to varying extents. Indeed, although nisin A M17Q followed by nisin A HTK appeared to be the most active variants as observed by deferred antagonism assays, in many cases, specific activity assays with purified peptides revealed this not to be the case. It is likely that the heightened bioactivity was as a result of enhanced diffusion in agar in a manner similar to that reported previously [20,30]. This attribute was noted as being beneficial in that one such peptide, the hinge derivative SVA, outperformed wild type nisin A in controlling *Listeria monocytogenes* in a model food system [30].

Notably, derivatives at threonine 2 and methionine 17 provided the first suggestion that nisin could be improved against particular targets when nisin Z T2S, and nisin Z M17Q/G18T demonstrated better activity against two non-pathogenic target strains (*M. flavus*, *S. thermophilus*) [36]. Additionally, investigations that involved the in vivo incorporation of tryptophan analogues into nisin in a bid to yield improved or altered-specificity variants revealed that M17W displayed 17-fold less activity than parental nisin A [23]. More recently, a study has demonstrated the enhanced specific activity of nisin A M17Q over nisin A against clinical strains of *S. epidermidis*, revealing it to be superior at reducing biofilm production on medical device substrates and at reducing bacterial numbers in a simulated wound fluid [37]. The contrasting sensitivity of mastitis-associated *S. aureus* and industrially relevant lactococci to nisin A M17Q, nisin A T2L and nisin A HTK provides additional data that some bioengineered nisin derivatives exhibit target-specific variations in potency. Indeed, the variation in specific activities may reflect the enormous wealth and lineages of *S. aureus* strains that differ markedly with regard to pleiotropic transcriptional regulators and the numerous two-component systems (TCS) that act as a sophisticated arsenal of environmental monitoring proteins [38]. For example, BraRS, the BceRS-like TCS associated with *S. aureus*, has been shown to be essential for resistance to bacitracin, nisin, and nukacin ISK-1 by permitting the cell to adapt and survive through co-ordinated gene expression and cell envelope modification [6]. Furthermore, *S. aureus* possess genetic systems (*dltABCD* and *mprF*) that bring about alterations of their cell envelope surface electrostatic properties, resulting in a shift in membrane charge and a subsequent repulsion of cationic antimicrobial peptides including nisin [39]. Moreover, studies have revealed that resistance to nisin evolves easily in *S. aureus* as a result of mutations within the *nsa*S gene, encoding the sensor kinase component of the NsaRS signal transduction system [40]. Such systems in *S. aureus* present a significant challenge to its effectiveness as an antimicrobial and careful consideration is required in the context of advancing nisin and other bacteriocins toward therapeutic exploitation.

One potential strategy to surmount this resistance problem could involve derivatization of nisin to counteract such systems in recognizing it as a substrate. For example, a recent study involving high resolution NMR studies of nisin in cellular membranes identified flexible domains that permit the lantibiotic to adapt to the cellular environment [41]. Intriguingly, these plastic domains corresponded to pharmaceutical hotspots identified previously, including Ile 4, Lys12, the hinge region and Ser29 [20,24,25,27,42], and enabled the specific activity of nisin to be enhanced, confirming a link between antimicrobial activity and cellular adaptability. The nisin A HTK peptide represents a novel hinge derivative to add to those previously identified through site-saturation mutagenesis of the hinge region, namely AAK, NAI and SLS, that displayed enhanced bioactivity against a variety of targets [20]. In particular, nisin A T2L and nisin A M17Q represent new derivatives whose locations were not deemed hotspots by NMR studies [41]. This contrasted with a report that revealed the derivative nisin Z T2S (which was modified to dehydroalanine) displayed a two-fold increase in specific activity against two non-pathogenic indicators, whilst T2A and T2V remained either fully or almost fully active respectfully when compared to the wild type [43]. Furthermore, in a recent study that used bioengineering to generate a chimeric lantibiotic composed of two lipid-binding motifs, i.e., those of nisin and the alpha peptide of the two-component haloduracin (HalA1), the importance of Thr 2 within the lipid II binding motif of nisin was highlighted when introduction of aspartate (T2D) completely abolished antimicrobial activity [44].

In addition to potent antimicrobial activity, nisin autoregulates its own production via a two-component signal transduction pathway. Numerous enquiries have applied site-specific or site-saturation mutagenesis to identify structural features or several specific sites of nisin in a bid to identify those that play a key role in this induction capacity [21,36,45]. With particular relevance to this study, it was previously shown that the variants nisin Z T2S and nisin Z M17W displayed 11-fold and 2-fold increases in induction capacity compared to the wild-type peptide, respectively [36]. Our investigations using a GFP reporter system revealed that nisin A T2L and nisin A M17Q induced promoter activity comparable to that of nisin A at the commonly used inducing concentration of 10 ng/mL. When a higher concentration of peptide was used, a significant delay in growth of the reporter strain was observed for nisin A, which was not apparent for nisin A T2L and nisin A HTK, providing further evidence of the attenuated antimicrobial activity of the variant peptides.

This study highlights the advantages of more methodical and rational screening strategies that generate molecular diversity through random and site saturation mutagenesis, followed by identification of library members with improvements by high-throughput screening or selection. In addition, the large dataset generated could potentially be evaluated through multidisciplinary approaches including quantum mechanical and molecular dynamics simulations as well as machine-learning algorithms to effectively explore the impact of amino acid substitutions on lantibiotic peptide structure and stability. For example, recent molecular dynamics simulations combined with microbiological techniques provided valuable insights at the atomic level into the interactions of nisin and an improved nisin derivative in association with the nisin resistance protein [28]. Additionally, recent studies have revealed new inhibitory mechanisms for nisin where in addition to membrane depolarization and rapid killing of cells, *S. aureus* strains exposed to nisin exhibited strong condensation of DNA, impeding chromosomal replication or segregation and suggests that this DNA damage might be a crucial component in the killing mechanism of nisin in *S. aureus* [46].

The use of nisin A HTK, nisin A M17Q and nisin A T2L peptides as therapeutics for the treatment of mastitis would have many benefits given that milk LAB, including adventitious (nonstarter; NSLAB) and introduced (starter cultures and adjuncts), are key to the generation of fermented dairy products. LAB instigate milk fermentation through lactate production and significantly influence the texture, consistency, taste and organoleptic properties of resultant products [47]. In addition, in contrast to antibiotics, the presence of nisin residues in milk may not require that the milk be withheld as nisin is susceptible to digestive enzymes and is easily destroyed in the gut. From a clinical perspective, nisin has already shown great promise in treating infectious mastitis in lactating mothers [48]. Notably, a significant reduction in staphylococcal numbers in breast milk was observed following treatment with nisin extracted from a *L. lactis* producer strain and no clinical signs of mastitis were apparent following two weeks of treatment. Crucially, nisin was effective where traditional antibiotic interventions failed to deliver any improvement [48]. Nisin A has been used commercially in the veterinary arena as anti-mastitis products in the form of pre-treated wipes to clean and disinfect the teat area prior to and post milking and also as an intramammary infusion product (Immucell Corp., Portland, ME, USA). Furthermore, it is anticipated that nisin will gain FDA approval in the US as a treatment for sub-clinical mastitis without the need to discard milk or withhold meat from the food chain following treatment (the first such designation given to any intramammary mastitis treatment product) [49]. Certainly, given the targeted potencies of nisin A M17Q, nisin A HTK and in particular nisin A T2L against the staphylococci and lactococci of this study, these peptides warrant further consideration as novel anti-mastitis antimicrobials.

## 4. Conclusions

*Staphylococcus aureus* is a major aetiological agent of bovine mastitis that often results in long-lasting, persistent and recurrent infections. Due to the rise in antibiotic resistance development and its potent efficacy against multi-drug resistant pathogens, there is a renewed interest in applying nisin as a chemotherapeutic to treat bacterial infections. Here, we report the screening of a large bank of bioengineered nisin derivatives and the subsequent identification of three novel variants, nisin A M17Q, nisin A HTK and nisin A T2L, that exhibit strain-specific enhanced potency against pathogenic staphylococci including bovine mastitis-associated strains and reduced activity against many of the commensal organisms that compose the milk microbiota such as lactococci and lactobacilli. In our opinion, these findings suggest that these bioengineered derivatives merit further investigation as novel antimicrobials in the treatment of bovine mastitis.

## 5. Materials and Methods

### 5.1. Bacterial Strains and Growth Conditions

*L. lactis* strains were grown in M17 broth (Oxoid, Waltham, MA, USA) supplemented with 0.5% glucose (GM17) or GM17 agar at 30 °C. Bifidobacteria were grown anaerobically at 37 °C in Reinforced Clostridial Medium (RCM) or RCM agar (Oxoid, Waltham, MA, USA), lactobacillus strains were grown in De Man Rogosa and Sharpe (Oxoid, Waltham, MA, USA) (MRS) or MRS agar anaerobically at 37 °C. *Escherichia coli* was grown in Luria-Bertani broth with vigorous shaking or agar at 37 °C. *Staphylococcus* and *Streptococcus* strains were grown in brain heart infusion (BHI) (Oxoid, Waltham, MA, USA) or tryptic soy broth (TSB) (Merck, Kenilworth, NJ, USA)at 37 °C. Antibiotics were used where indicated at the following concentrations: Chloramphenicol (Sigma, St. Louis, MO, USA) at 10 and 20 μg mL^−1^ for *L. lactis* and *E. coli*, respectively.

### 5.2. Generation and Assessment of a Bank of Nisin Derivatives

Mutagenesis of the *nis*A gene was carried out as described previously [24,25]. Deferred antagonism agar-based assays were employed to assess the bioactivity of nisin derivative-producing strains. Briefly, the *L. lactis* producers were ‘spotted’ (approximately 3 µL) onto GM17 agar using a 96-pin microplate replicator (Boekel, Feasterville, PA, USA) and incubated for 16 h at 30 °C. Growth media (0.75% agar) suitable for growth of the individual target strains (staphylococci, streptococci, lactococci, etc.) was seeded (0.5%) and poured over the *L. lactis* producers followed by further incubation at conditions suitable for the indicator. Enhancement in bioactivity was indicated by increased zone of inhibition relative to that generated by the wild-type producer. Conversely, reduced bioactivity was signified by reduced zones of inhibition compared to the wild type producer. Nisin producers of interest (nisin A HTK, nisin A M17Q and nisin A T2L) were spotted in triplicate and overlaid with 3 separate cultures of each of the test strains. Zones of inhibition in the overlaid agar were measured using Vernier calipers, recorded in millimeters and rounded to two decimal places.

### 5.3. Identification of Nisin Derivatives

The changes to the nisA genes within the corresponding pDF05 (pCI372-nisA) [24] derivatives were established through DNA sequencing (SourceBioscience, Waterford, Ireland) using the primers pCI372For 5′-CGGGAAGCTAGAGTAAGTAG-3’ and pCI372Rev 5′-ACCTCTCGGTTATGAGTTAG-3′. Sequence alignments with the nisA gene were carried out with Lasergene Megalign 7.00 (DNAStar) to determine the nature of the codon changes.

### 5.4. Mass Spectrometry

Colony mass spectrometry (CMS) of *L. lactis* transformants was performed by mixing cells with 50 µL of 70% isopropanol (Fisher Scientific, Waltham, MA, USA) containing 0.1% TFA. The cell suspension was vortexed, centrifuged at 14,000 r.p.m. for 2 min, and the supernatant recovered for analysis. In all cases mass spectrometry was performed with an Axima TOF^2^ MALDI TOF mass spectrometer (Shimadzu Biotech, Manchester, UK). Matrix solution (alpha-cyano-4-hydroxy cinnamic acid (CHCA), 10 mg mL^−1^ in 50% acetonitrile 0.1% (*v*/*v*) trifluoroacetic acid) was positioned onto the target for 60 s and then removed. The remaining solution was then allowed to air-dry and the sample solution (purified peptide resuspension or CMS supernatant) was placed onto the precoated sample spot. Following addition of 0.5 µL matrix solution and air-drying, the sample was subsequently analysed in positive-ion reflectron mode.

### 5.5. Nisin Purification

Strains producing peptides of interest were inoculated (1% fresh overnight growth) into 2 litres of tryptone yeast (TY) (Merck, Kenilworth, NJ, USA) broth to which was added glucose (0.5% *v*/*v*) (Sigma-Aldrich, St. Louis, MO, USA) and ß-glycerophosphate (Sigma-Aldrich, St. Louis, MO, USA) (2% *v*/*v*) and incubated for 16–18 h. The sample was then centrifuged for 20 min at 8630 g. The cell free supernatant (CFS) was passed through 60 g of pre-equilibrated Amberlite XAD16 beads (Sigma-Aldrich, St. Louis, MO, USA) and washed with 500 mL of 30% ethanol and eluted in 500 mL of 70% isopropanol (IPA) (Fisher Scientific, Waltham, MA, USA) with 0.1% trifluoroacetic acid (TFA) (Sigma-Aldrich, St. Louis, MO, USA). Concomitantly, the cell pellets were resuspended in 300 mL of 70% IPA 0.1% TFA and stirred for 3 h at room temperature and then centrifugation was applied as above. The supernatants from above were concentrated through rotary-evaporation (Buchi, Flawil, Switzerland) to approximately 250 mL, adjusted to pH 4.0 and passed through a Phenomenex SPE C-18 column to a final volume of 60 mL. Twelve milligrams was further concentrated by rotary evaporation to 2 mL and purified through HPLC using a Phenomenex C12 Reverse-Phase (RP) HPLC column (Jupiter 4 µ proteo 90 Å, 250 × 10.0 mm, 4 µm) in a gradient of 25–60% acetonitrile (Fisher, Waltham, MA, USA) containing 0.1% TFA. The relevant fractions were collected and pooled and freeze-dried (LABCONCO, Kansas City, MO, USA). The purified peptides were subjected to MALDI-TOF Mass Spectrometry analysis to confirm their purity before use.

### 5.6. Minimum Inhibitory Concentration Assays

Minimum inhibitory concentration determinations were carried out as described previously [26]. Briefly, 96 well microtiter plates were pre-treated with bovine serum albumin (BSA) (Sigma-Aldrich, St. Louis, MO, USA). Purified wild type and derivative peptides were resuspended in the appropriate media and added to the first well to 7.5 or 5.0 μM or 500 nM starting concentration. Subsequently, 2-fold serial dilutions of each peptide were made in 96 well plates for a total of 12 dilutions. Each target strain was subcultured and incubated to reach an OD 600 nm of 0.5 and diluted to 10^5^ cfu/mL and added to each test well. The plates were incubated at the appropriate temperature (37 °C or 30 °C) for 16 h and the MIC read as the lowest peptide concentration where growth was not visible.

### 5.7. Growth Curve Analysis

For growth curve assays, fresh overnight cultures were transferred (10^6^ cfu mL^−1^ in a volume of 1.0 mL) into BHI broth (staphylococci) or GM17 broth (lactococci) to which had been added nisin A, HTK, M17Q, and T2L peptides at increasing concentrations (i.e., 50, 100, 150, 200 nM, etc.). Subsequently 0.2 mL was transferred to 96 well microtitre plates (Sarstedt, Newton, NC, USA) and cell growth was measured spectrophotometrically over 24 h periods by using a SpectraMax M3 spectrophotometer (Molecular Devices, Sunnyvale, CA, USA). Experiments were carried out in triplicate.

### 5.8. GFP Assays

The induction capacity of native nisin A and its derivatives was assessed using a GFP reporter assay following the method previously described [28] but with modifications. Briefly, cells from a fresh overnight of *L. lactis* NZ9000pNZ8150gfp+ were diluted in fresh GM17 to reach a final concentration of 10^6^ CFU/mL. Peptides of nisin A, HTK, M17Q and T2L were added to reach a final concentration of 10 and 30 ng/mL. Next, 2 mL was transferred to 24 well microplates with black well walls and clear bottom (Perkin Elmer, Waltham, MA, USA) to enable bottom read fluorescence measurements. Additionally, 0.2 mL was transferred to a flat bottom 96-well microtiter plate (Sarstedt, Newton, NC, USA) to enable concomitant absorbance readings. Green Protein Fluorescence was monitored with a SpectraMax M3 spectrophotometer (Molecular Devices, Sunnyvale, CA, USA) as Relative Fluorescence Units (RFU) with excitation and emission filters set at 485 nm and 528 nm, respectively. Absorbance readings were taken at OD_595_ using a Multiskan FC microplate photometer, v1.01.14 (Thermo Scientific, Waltham, MA, USA). The baseline fluorescence and absorbance of uncultured media was subtracted from all subsequent readings using SoftMax Pro v6.3 and SkanIt RE v4.1 software, respectively. Tests were carried out in triplicate.

### 5.9. Statistical Analysis

Statistical analysis was carried out in GraphPad Prism 5.03 software following tests for normality and homogeneity of variances (Levene’s test) in R (R Core Team 2020). Parametric data were analyzed by the independent Student *t* test. Nonparametric data were analyzed by the Mann–Whitney *U*-test. Statistical significance was defined as having a *p* value of <0.05.

## Figures and Tables

**Table 1:** Bioactivity of nisin and nisin variant producers against representative bovine-mastitis associated strains and dairy lactococci strains. Values given are the mean of triplicate deferred antagonism assays where the diameter of the zone of inhibition is in mm. Asterisk denotes statistical significance compared to nisin A (*p* < 0.05).

Indicator	Nisin A (mm)	HTK (mm)	M17Q (mm)	T2L (mm)
*S. aureus* RF122	10.44 ± 0.69	14.89 ± 0.47 *	15.39 ± 1.7 *	10.15 ± 0.11
*S. aureus* NCDO1499	11.73 ± 0.54	14.75 ± 0.48 *	16.51 ± 0.16 *	10.20 ± 0.13 *
*S. aureus* DPC 5243	12.71 ± 0.33	16.06 ± 0.37 *	16.76 ± 0.55 *	10.04 ± 0.43 *
*S. aureus* ST528 (MRSA)	12.25 ± 0.30	14.91 ± 0.26 *	16.32 ± 1.2 *	10.09 ± 0.64 *
*S. aureus* ST534 (MRSA)	7.51 ± 0.19	10.41 ± 0.88 *	12.17 ± 0.36 *	7.26 ± 0.66
*S. aureus* SA113	6.90 ± 0.16	8.34 ± 0.57 *	9.14 ± 0.51 *	6.28 ± 0.08
*S. aureus* SA113 mprf	10.16 ± 0.52	13.34 ± 0.25 *	12.98 ± 0.53 *	7.83 ± 0.1 *
*S. aureus* SA113 *dltA*	13.09 ± 0.84	15.66 ± 0.54 *	17.51 ± 1.40 *	12.75 ± 0.75
*S. uberis* DPC5344	17.59 ± 0.37	18.66 ± 0.69	18.27 ± 0.47	16.93 ± 1.50
*S. dysgal* ATCC43078	10.55 ± 0.48	15.51 ± 0.27 *	11.99 ± 0.47 *	11.28 ± 0.32 *
*S. agalactiae* ATCC13813	9.34 ± 0.15	9.36 ± 0.08	12.25 ± 0.11 *	9.08 ± 0.12
*L. lactis* MG1363	9.39 ± 0.04	9.12 ± 0.13	11.25 ± 0.24 *	7.54 ± 0.39 *
*L. lactis* HP	23.65 ± 0.13	23.17 ± 0.42	27.69 ± 0.70 *	20.33 ± 0.66 *
*L. lactis* KH	17.43 ± 1.89	17.71 ± 2.03	20.36 ± 2.2	14.72 ± 2.06
*L. lactis* IP5	17.14 ± 2.2	18.13 ± 3.1	20.71 ± 2.2	14.13 ± 3.2
*Lb. acidophilus ATCC 4356*	20.17 ± 0.25	17.75 ± 3.5	27.54 ± 1.2 *	17.43 ± 2.0 *

**Table 2:** Specific activity of nisin A, nisin A M17Q, nisin A T2L and nisin A HTK against a range of indicator organisms. Grey shading denotes a favourable outcome where specific activity is enhanced against staphylococci and reduced against a selection of lactic acid bacteria/bifidobacteria. No shade denotes no change. No shade/asterisk denotes unfavourable outcome.

Indicator	Nisin A µg/mL (µM)	HTK µg/mL (µM)	M17Q µg/mL (µM)	T2L µg/mL (µM)
*S. aureus* NCDO1499	2 (0.625)	1 (0.312)	1 (0.312)	0.5 (0.156)
*S. aureus* RF122	1 (0.312)	0.12 (0.039)	0.25 (0.078)	0.25 (0.078)
*S. aureus* DPC 5243	1 (0.312)	1 (0.312)	2 (0.625) *	0.5 (0.156)
*S. aureus* DPC 5245	1 (0.312)	1 (0.312)	1 (0.312)	0.5(0.156)
*S. aureus* DPC 5247	1 (0.312)	1 (0.312)	1 (0.312)	0.5(0.156)
*S. aureus* SA113	8 (2.5)	8 (2.5)	8 (2.5)	1 (0.312)
*S. aureus* SA113Δmprf	4 (1.25)	4 (1.25)	4 (1.25)	0.25 (0.078)
*S. aureus SA113*ΔdltA	2 (0.625)	2 (0.625)	2 (0.625)	0.06 (0.019)
*S. aureus* 513	6 (1.875)	6 (1.875)	6 (1.875)	3 (0.937)
*S. aureus* 272	3 (0.937)	6 (1.875) *	1.5 (0.468)	0.75 (0.234)
*S. aureus* ST528(MRSA)	0.5 (0.156)	0.25 (0.078)	0.25 (0.078)	0.125 (0.039)
*S. aureus* ST534(MRSA)	1 (0.312)	1 (0.312)	1 (0.312)	0.25 (0.078)
*S. dysgalactiae* ATCC43078	4 (1.25)	4 (1.25)	8 (2.5) *	8 (2.5) *
*L. lactis* MG1363	0.2 (0.06)	0.1 (0.03) *	0.1 (0.03) *	0.2 (0.06)
*L. lactis* HP	0.2 (0.06)	0.4 (0.125)	0.1 (0.03) *	0.4 (0.125)
*L. lactis KH*	0.05 (0.015)	0.05 (0.015)	0.05 (0.015)	(0.031)
*L. lactis* IP5	0.25 (0.078)	0.25 (0.078)	0.25 (0.078)	1 (0.312)
*B. longum* UCC 44b	0.06 (0.019)	0.24 (0.072)	0.06 (0.019)	0.12 (0.039)
*Lb. acidophilus ATCC 4356*	0.03 (0.010)	0.12 (0.039)	0.06 (0.022)	0.12 (0.039)

## Data Availability

The data presented in this study are available on request from the corresponding author.

## Supplementary Materials

The following are available online at https://www.mdpi.com/article/10.3390/ijms22073480/s1, **Figure S1**: RP-HPLC profiles and MALDI-ToF mass spectrometric (MS) analysis (inset) of (**A**) nisin A HTK (**B**) nisin A M17Q, (**C**) nisin A T2L, (**D**) nisin A (wild type).
