# Supplementary material for: Bio-Engineered Nisin with Increased Anti-Staphylococcus and Selectively Reduced Anti-Lactococcus Activity for Treatment of Bovine Mastitis
Source: Int J Mol Sci. 2021 Mar 27;22(7):3480. doi: 10.3390/ijms22073480 (PMC8036683; doi:10.3390/ijms22073480)
Supplement: Supplementary file 1 [file ijms-22-03480-s001.pdf]

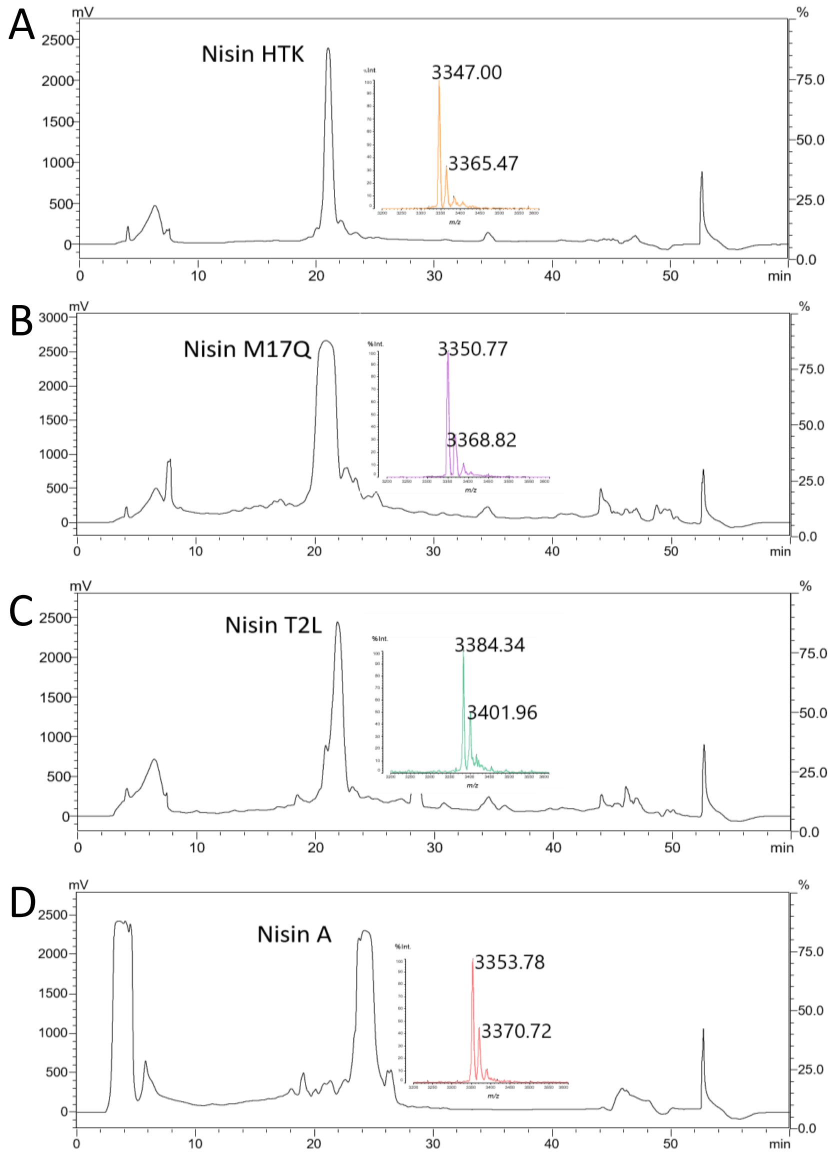

Figure S1: RP-HPLC profiles and MALDI-ToF mass spectrometric (MS) analysis (inset) of (**A**) nisin A HTK (**B**) nisin A M17Q, (**C**) nisin A T2L, (**D**) nisin A (wild type).
